# Asymmetric Dimethylarginine (ADMA) in Pediatric Renal Diseases: From Pathophysiological Phenomenon to Clinical Biomarker and Beyond

**DOI:** 10.3390/children8100837

**Published:** 2021-09-24

**Authors:** Chien-Ning Hsu, You-Lin Tain

**Affiliations:** 1 Department of Pharmacy, Kaohsiung Chang Gung Memorial Hospital, Kaohsiung 833, Taiwan; cnhsu@cgmh.org.tw; 2 School of Pharmacy, Kaohsiung Medical University, Kaohsiung 807, Taiwan; 3 Department of Pediatrics, Kaohsiung Chang Gung Memorial Hospital, Chang Gung University College of Medicine, Kaohsiung 833, Taiwan; 4 Institute for Translational Research in Biomedicine, Kaohsiung Chang Gung Memorial Hospital, Chang Gung University College of Medicine, Kaohsiung 833, Taiwan

**Keywords:** asymmetric dimethylarginine, biomarker, dimethylamine, kidney disease, nitric oxide, children, dimethylarginine dimethylaminohydrolase, protein arginine methyl transferase, pediatric nephrology

## Abstract

Asymmetric dimethylarginine (ADMA), an endogenous nitric oxide (NO) synthase inhibitor, inhibits NO synthesis and contributes to the pathogenesis of many human diseases. In adults, ADMA has been identified as a biomarker for chronic kidney disease (CKD) progression and cardiovascular risk. However, little attention is given to translating the adult experience into the pediatric clinical setting. In the current review, we summarize circulating and urinary ADMA reported thus far in clinical studies relating to kidney disease in children and adolescents, as well as systematize the knowledge on pathophysiological role of ADMA in the kidneys. The aim of this review is also to show the various analytical methods for measuring ADMA and the issues tht need to be addressed before transforming to clinical practice in pediatric medicine. The last task is to suggest that ADMA may not only be suitable as a diagnostic or prognostic biomarker, but also a promising therapeutic strategy to treat pediatric kidney disease in the future.

## 1. Introduction

Asymmetric dimethylarginine (ADMA) is a naturally occurring amino acid [1]. ADMA has received much attention over the past decades as it is an endogenous inhibitor of nitric oxide (NO) production [2,3,4]. ADMA is involved in the pathogenesis of a wide spectrum of human diseases [1]. According to current evidence, ADMA has been considered as a biomarker predicting higher mortality in chronic kidney disease (CKD) [5], as well as a faster progression of kidney injury [6]. However, much less attention has been paid to studying ADMA in the pediatric population. There has been growing research interest in ADMA with a thorough investigation as updated on July 2021, yielding more than 3000 items in PubMed. However, only less than 10% of the articles are related to pediatrics. Thus, the focus of the current review is on the clinical significance of ADMA in pediatric renal diseases from a clinician’s perspective, beyond pathophysiological phenomena. Our search strategy was designed to retrieve literature relating to ADMA from PubMed/MEDLINE databases. Specific emphasis was put on clinical studies on neonates, children, and adolescents reporting on ADMA and related NO parameters in the renal system. Additional studies targeting the pathophysiological phenomenon of ADMA related to its clinical significance were also considered.

## 2. ADMA: A Historical Perspective

In 1970, long before the discovery of NO, Kakimoto and Akazawa first identified and isolated ADMA from human urine [7]. Though renal excretion was initially considered as the major route for ADMA removal, a study from McDermott suggested ADMA may undergo extensive metabolism [8]. Ogawa et al. further identified the enzyme dimethylarginine dimethylaminohydrolase-1 (DDAH-1) that metabolizes ADMA to generate L-citrulline and dimethylamine (DMA) in 1987 [9]. In 1999, a second DDAH isoform (DDAH-2) was discovered [10]. A newly discovered mitochondrial aminotransferase expressed primarily in the kidney, namely alanine-glyoxylate aminotransferase 2 (AGXT2), can also metabolize ADMA [11].

The biologically relevant effects of ADMA as an NO synthase (NOS) inhibitor were first reported by Vallance and colleagues [2]. In 1992, they showed that ADMA can inhibit NO synthesis and also demonstrated that hemodialysis patients had higher blood ADMA levels than controls [2]. According to these findings, the possibility of ADMA acting as endogenous regulators in the NO pathway in health and diseases raised considerable interest. So far, there has been mounting evidence showing that ADMA is involved in the pathophysiology of diverse biological functions, including endothelial dysfunction [3], apoptosis, [12], oxidative stress [13], autophagy [14], gene regulation [15,16], inflammation [17], and immunological function [18]. Overall, ADMA now has a significant impact on human health and potential therapeutic strategies [4,19,20,21,22].

### 2.1. ADMA Biosynthesis and Metabolism

Methylarginines are continuously produced by protein methylation during normal protein turnover. Arginine residues in proteins are methylated by a family of protein arginine methyl transferases (PRMTs) to form protein-bound ADMA. Free ADMA is created on proteolysis of methylated proteins [23]. Symmetric dimethylarginine (SDMA) is a structural isomer of ADMA. Today, it is clear that type I PRMTs (PRMT-1, -3, -4, -6, and -8) produce ADMA, while type II PRMTs (PRMT-5 and -9) generate SDMA [23].

After release, free ADMA migrates into the extracellular space and circulation. Free ADMA and SDMA share a common transport process. The cationic amino acid transporter (CAT) family can transport ADMA in and out of cells [24]. CATs mediate uptake of ADMA by neighboring cells or distant organs, thereby promoting active interorgan transport. Free ADMA can be transported through circulation into target organs such as the kidney for enzymatic degradation. ADMA is eliminated partly via urinary excretion but mainly via metabolism. A healthy adult produces 60 mg of ADMA per day (~300 μM), of which around 10–20% is excreted in urine via the kidneys [25].

Today, three enzymes have been identified to degrade ADMA: DDAH-1, DDAH-2, and AGXT2 [9,10,11]. The majority of ADMA involves its hydrolysis to DMA and L-citrulline by DDAHs. In addition, ADMA can also be transaminated by AGXT2 to form α-keto-δ-(N^G^,N^G^-dimethylguanidino) valeric acid (DMGV) [11]. ADMA concentrations in the plasma and tissues, hence, are dependent on factors that can inhibit DDAHs [26], including hyperglycemia [27], angiotensin II administration [28], and oxidative stress [29].

The main biologic action of ADMA is the inhibition of NO biosynthesis. At physiological conditions, NOS is well saturated with the substrate L-arginine and NO is generated. When intracellular ADMA reaches the pathological concentration, it competes with L-arginine and thus reduces NO production. Under such conditions, the addition of exogenous L-arginine displaces ADMA intracellularly and restores the physiological L-arginine-to-ADMA ratio to a level enough to restore NO production [25]. Accordingly, NO biosynthesis depends on the local L-arginine-to-ADMA ratio.

**Figure 1:**
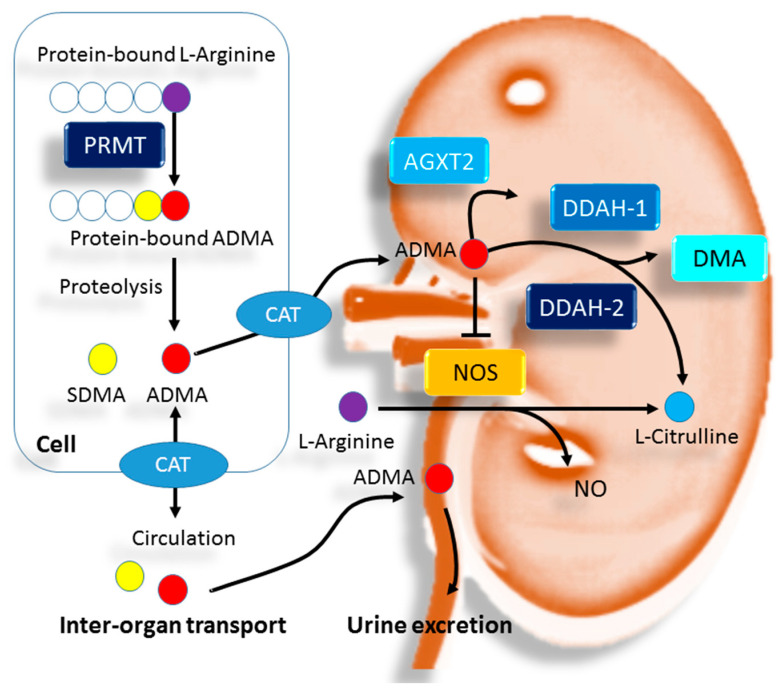
Simplified schema of synthesis, transport, and elimination of ADMA in the kidney. The enzymes in protein arginine methyltransferases (PRMTs) family methylate protein-bound L-Arginine residues (purple cycle) to generate protein-bound ADMA (red circle) and SDMA (yellow circle). Upon proteolysis, free ADMA is released and moved out of the cells via cationic amino acid transporter (CAT). In the kidneys, ADMA can be removed via urinary excretion or enzymatic degradation. Dimethylarginine dimethylaminohydrolase-1 (DDAH-1) and -2 (DDAH-2) can catalyze ADMA to generate L-Citrulline and dimethylamine (DMA). In addition, ADMA can be metabolized by alanine-glyoxylate aminotransferase 2 (AGXT2). In the kidney, ADMA can inhibit nitric oxide (NO) synthase to inhibit NO production.

Intracellular ADMA levels can be 5- to 20-fold higher than those in the plasma, in an organ-specific manner [24]. This discrepancy of ADMA concentration across different organs can be the result of differential expression of DDAHs in different organs. Data from animal research indicated that the concentrations of ADMA were highest in the kidney, liver, spleen, and pancreas, followed by the heart and lung, and lowest in the brain [30]. DDAH-1 deficient mice showed that ADMA is mainly regulated by DDAH-1, which is highly expressed in the liver and kidney cortex, the main sites of ADMA metabolism [31]. These findings suggest that both the kidney and the liver are major sites for the metabolism of excessive circulating ADMA [32]. The biosynthesis and elimination of ADMA and the relation of ADMA to NO are illustrated diagrammatically in Figure 1.

### 2.2. Quantification of ADMA

As ADMA has a narrow range of normal concentrations, a high-precision analytical method is required to distinguish between normal and slightly high levels [33]. To date, several analytical methods for the quantitative determination of ADMA concentrations include high-performance liquid chromatography (HPLC), HPLC with mass spectrometric detection (HPLC–MS) [34], ultrahigh performance liquid chromatography (UPLC)–MS/MS [35], liquid chromatography (LC)–MS and LC–MS/MS [36,37], gas chromatography (GC)–MS [38], and enzyme-linked immunosorbent assay (ELISA) [39].

In clinical and experimental studies, HPLC-based methods are the most commonly used techniques for determining ADMA concentrations in the plasma, urine, and tissue homogenate [34]. Since ADMA and its structural isomer SDMA have identical molecular weights, chromatographic separation using HPLC with radioimmunoassay, fluorescence (FL), or ultraviolet (UV) detection was shown to be mandatory [40]. Among them, fluorescent derivatization with ortho-phthaldialdehyde (OPA) or AccQ-Fluor has been the most frequently used HPLC method for measuring plasma and tissue ADMA [40]. However, the required time consumption is a major concern for these HPLC methods.

Prior research has noted variation in ADMA levels between laboratories using different analytical methods. As reviewed elsewhere [40], circulating ADMA levels of healthy adults reported by different groups of investigators show a diverse range from 0.12 to 4.0 µM/L. Techniques utilizing the specificity of MS-based methods report mean values in healthy adults between 0.12 and 1.34 µM/L ADMA. Although MS-based methods are more sensitive, these techniques require considerably more expensive instrumentation that may be out of reach for most hospitals on a routine basis. An ELISA method used for determining plasma/serum ADMA has also been developed. However, it tends to overestimate ADMA concentrations [41]. Additionally, most studies showed a poor correlation between quantification by ELISA compared with other methods for determining ADMA [40,41,42]. Importantly, standardized analytical methods with sufficient sensitivity and specificity as well as reproducibility will be essential for ADMA to be reliably assessed on a routine basis in clinical space.

In addition to analytic methods, the variability in ADMA concentrations may be attributed to age. In adults, plasma ADMA levels increase with age. The mean concentration for a healthy adult is between 0.4 and 0.6 µM/L, with an approximately two-fold increase in the geriatric population [43]. There seems no sex difference exists in ADMA concentration [31,40]. On the other hand, ADMA levels are higher in the pediatric population than in adults. In neonates, ADMA values in venous cord blood are significantly higher (~1.06 µM/L) and drop with a mean declining rate of 15 nM per year from birth until near the age of 25 years [44,45]. Accordingly, these observations demonstrated a U-shaped relationship between blood ADMA levels and age, with the highest values in elderly and young children. It is noteworthy, however, that many human diseases are related to plasma levels of ADMA [1], its tissue level remains largely unknown in clinical studies.

### 2.3. ADMA and Kidney

Kidneys perform crucial functions in ADMA metabolism; they excrete ADMA and express high levels of DDAH to metabolize ADMA. As ADMA is listed as uremic toxins by the European Uremic Toxin Work Group (EUTox) [46], there is a growing demand from clinicians to better understanding levels of ADMA for kidney diseases.

Considering ADMA has a low molecular weight similar to urea, dialysis is considered the ideal option for its removal of ADMA [47]. However, it was shown that a single dialysis session reduced ADMA levels by 23% [48]. After dialysis, a rebound increase in plasma ADMA levels can reach an even higher level compared to the baseline. Increased ADMA levels in both patients with CKD and end-stage kidney disease (ESKD) are reported in many studies, as reviewed elsewhere [6,49,50]. Plasma ADMA levels may predict the progression of kidney injury and cardiovascular risk and mortality in patients with CKD [5,6,50].

At least three possibilities exist for an elevation of plasma ADMA: a decrease in renal excretion, a decreased enzymatic metabolism, and an increased synthesis of ADMA. The first two mechanisms have been shown to contribute to elevations of ADMA in kidney disease, whereas the impact of PRMT-mediated increased synthesis remains unknown [49]. Although ADMA is excreted by the kidneys to some extent, decreased ADMA metabolism is the major reason for its elevation in kidney disease.

In the kidney, ADMA can regulate NO and therefore govern many important functions. These include regulation of renal hemodynamics, mediation of pressure-natriuresis, modulation of medullary blood flow, modulation of renal sympathetic neural activity, blunting of tubuloglomerular feedback, regulation of BP, and inhibition of sodium reabsorption [51,52].

In the absence of human data, research with experimental animals is the most reliable means of exploring the role of ADMA in tissues. In spontaneously hypertensive rats (SHRs), elevated ADMA levels in the kidneys and lungs have been reported [53,54]. Additionally, ADMA concentrations are increased in the aortas and kidneys of diabetic rodents [55,56]. These findings support a proposed role for tissue ADMA in various diseases. In a young rat bile-duct ligation (BDL) model, we simultaneously determined ADMA concentrations in the plasma, liver, and kidneys [57]. We found that increases in ADMA in the plasma are largely due to increased synthesis of ADMA coinciding with enhancing PRMT1 abundance in the liver. Although the metabolism of ADMA is unaltered in the damaged liver, decreased renal DDAH activity resulting in the kidneys are unable to metabolize excessive ADMA. We also found that ADMA levels in the brain cortex of young BDL rats were unaltered, unlike in the liver and kidneys [58]. Thus, results from these studies that changes in plasma ADMA do not always correlate with tissue ADMA levels. There will be a growing need to be able to analyze tissue ADMA levels and better understand the impact of tissue ADMA levels apart from plasma ADMA levels in clinical research.

Furthermore, our previous report showed that ADMA can impair developing kidneys, resulting in reduced nephron number [56]. Metanephroi grown in 2 or 10 µM ADMA were found to have fewer and smaller nephrons in a dose-dependent manner [50]. Using next-generation RNA sequencing (NGS) analysis, we found that 1221 differential expressed genes were significantly altered in metanephroi treated with ADMA at the concentration of 10 µM [59]. Among them, *Avpr1a, Hba2, Hba-a2, Ephx2,* and *Npy1r* have been identified as differentially expressed genes in the kidney related to the regulation of BP [60]. An implication of these findings is ADMA plays a significant role in the development and function of the kidney.

## 3. ADMA as a Biomarker in Pediatric Kidney Disease

Table 1 summarizes the plasma and urinary ADMA levels, as well as their analytic methods in pediatric kidney disease, as reported in the literature. In 28 children and adolescents with CKD stage 2–3 and a mean age of 12.6 years, mean plasma ADMA levels were measured using HPLC–MS technique to be 1.1 µM/L, which was slightly higher than 0.8 μM/L in healthy controls [61,62]. Additionally, plasma ADMA levels were positively correlated with BP load.

**Figure 2:**
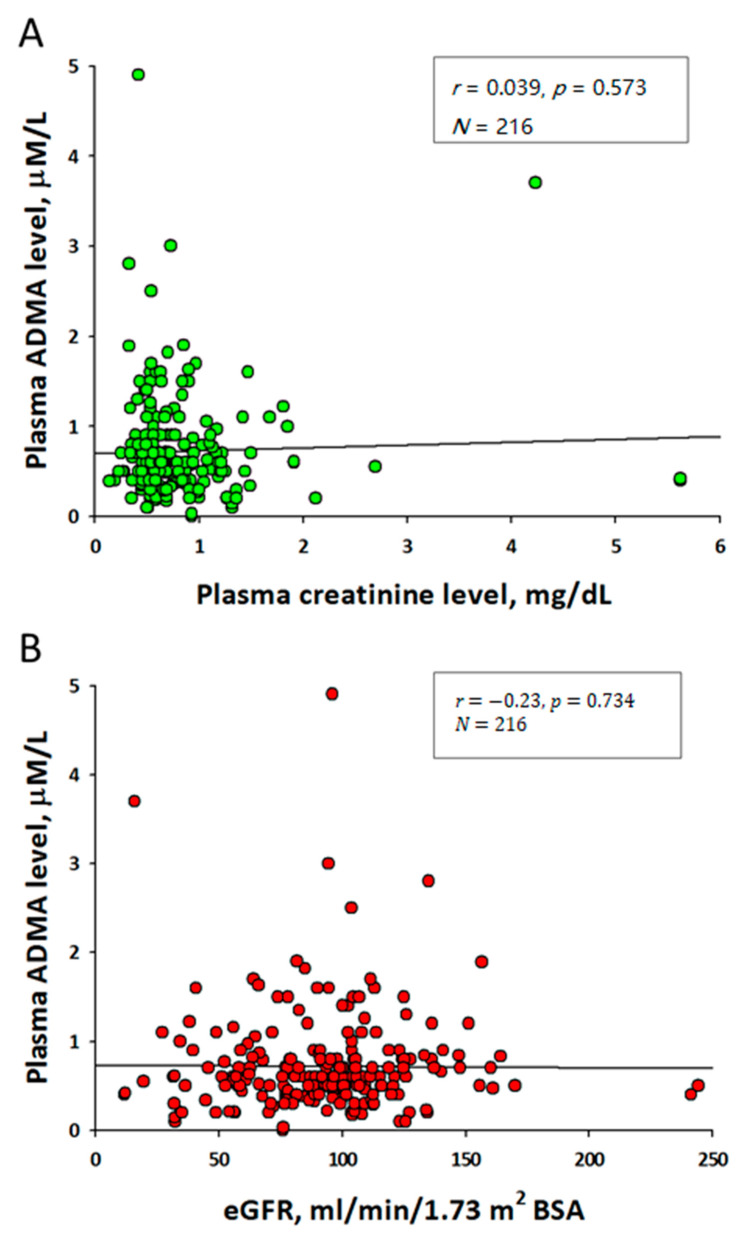
Person correlation and linear regression analyses between plasma ADMA level and (**A**) plasma creatinine level and (**B**) estimated glomerular filtration rate (eGFR) in 216 CKD stage 1–4 children and adolescents. This figure was constructed with data reported in part elsewhere [63,64,72,73].

In studies from our group, we used HPLC with fluorescence detection of OPA/3-mercaptopropionic acid (3MPA) derivatives to measure ADMA. In 57 children and adolescents with early stages of CKD, we found comparable plasma ADMA levels between CKD stage 1 and stages 2–3 [63]. There was a positive correlation between ADMA and augmentation index (AI), an arterial stiffness parameter. In 121 CKD stages 1–4 children who were normotensive or hypertensive, the corresponding median values of ADMA were 1.05 and 1.1, with no statistical difference between the two groups [64]. In adults, prior research demonstrated a gradual increase in ADMA concentrations with declining renal function after stratification for estimated glomerular filtration rate (eGFR) [49]. Since most pediatric studies are of limited values since they were either small sample size or use of different methods for measuring ADMA, it is hard to summarize available data to interpret whether ADMA correlates with renal function in pediatric CKD. We, therefore, pooled data from our previous studies in pediatric CKD using the same HPLC method to analyze ADMA and illustrated our results in Figure 2. Plasma ADMA level is not correlated with blood creatinine level or eGFR in children and adolescents with CKD, most likely because most ADMA undergoes enzymatic degradation.

In another small group of children (*n* = 32) with CKD stage 2–5, the median ADMA level in the plasma were 0.9 µM/L for CKD children and 0.7 µM/L for controls [65]. However, the increased ADMA with disease severity did not reach significance. A study that recruited 36 CKD and 40 ESKD children revealed that plasma ADMA levels were higher in those with ESKD than those with CKD [66]. Using the ELISA method, plasma ADMA concentrations were much higher in CKD children (0.65 ± 0.03 µM/L) compared to controls (0.39 ± 0.01 µM/L), with the highest values in ESKD children received hemodialysis (0.85 ± 0.01 µM/L) and peritoneal dialysis (0.78 ± 0.01 µM/L). Furthermore, another study of pediatric CKD demonstrated that the median ADMA level in the plasma was measured using ELISA to be 0.67 µM/L in healthy controls [67]. Nevertheless, ADMA concentrations of the CKD Stages 1–5 group were expressed as a z-score. The ADMA z-score was only higher than the control group in CKD Stage 5 children [67]. Though the use of z-scores in pediatrics is widespread to accurately assess growth through anthropometric measurements, calculating ADMA z-score might be inappropriate because of lacking normal reference values from the pediatric population.

As shown in Table 1, there were three reports investigating ADMA regarding pediatric glomerular kidney disease [68,69,70]. In 9 children with sporadic focal segmental glomerulosclerosis (FSGS) and 11 non-FSGS kidney diseases, the mean ADMA was measured by the GC–MS/MS method to be 0.85 ± 0.11 and 0.79 ± 0.13 µM/L, respectively [68]. This study demonstrated that plasma ADMA levels were only higher in FSGS but not in non-FSGS children compared to controls (0.68 ± 0.11 µM/L). In 32 children with idiopathic nephrotic syndrome (INS), differences in plasma ADMA values determined by the HPLC method at the relapse phase and remission were comparable [69]. These findings indicate that ADMA might not be a disease activity marker in childhood INS. Another study recruited 80 children with glomerular kidney disease and found that plasma ADMA levels were not different between patients with INS (1.72 ± 1.24 µM/L) and IgA nephropathy (IgAN)/Henoch-Schoenlein nephropathy (HSN) (1.6 ± 1.19 µM/L).

One study evaluated ADMA in pediatric hemolytic uremic syndrome (HUS), a frequent cause of acute renal failure in childhood. In 12 children with HUS who received PD, the mean ADMA levels were measured by the GC–MS/MS method to be 0.67 µM/L for HUS and 0.75 µM/L for controls [71].

In addition to testing plasma, there were four studies evaluating urinary ADMA concentrations in pediatric kidney disease [68,71,72,73]. In clinical studies, the collection of urine for 24 h is not always feasible in the pediatric population. Thus, urinary ADMA level is a correction of creatinine level using a spot urine sample. In pediatric CKD studies from our group [72,73], we found urinary ADMA level alone was not correlated with cardiovascular risks and CKD disease severity. However, the combined ratio between ADMA and other L-arginine metabolites, such as DMA and SDMA, provides a better correlation with BP load in children with early stages of CKD. In children with FSGS [68], elevated ADMA concentrations were found in plasma but not urine, presumably resulting from enhanced ADMA synthesis, suggesting a role of ADMA in the pathophysiology of FSGS. Another study demonstrated that both urinary and plasma ADMA concentrations were lower in children with HUS who received PD than controls [71]. Whether ADMA was removed by PD or a decreased synthesis of ADMA in these children awaits further evaluation. As ADMA can be degraded to DMA, the DMA level has been proposed as a measure of whole-body ADMA synthesis [74]. The DMA-to-ADMA ratio has also been proposed to reflect DDAH activity [75]. Our previous study showed that children with CKD stages 2–4 had higher plasma levels of DMA compared to those with CKD stage 1 [75]. Of note, DMA not only comes from ADMA but also a metabolic product of uremic toxin trimethylamine N-oxide (TMAO). Furthermore, urinary DMA levels could be seriously affected without dietary restriction as fish and seafood are abundant sources of DMA. Accordingly, measurements of the ADMA, DMA, and TMAO simultaneously warrant further investigations to explore the interplay between NO and TMAO pathways in pediatric kidney disease.

Considering the data in Table 1, plasma levels of ADMA in healthy children diverge from the studies using various analytical methods and different age populations. Similar to adults, children with ESKD had higher ADMA levels than those with early stages of CKD [66]. While data on ADMA correlating with disease severity in CKD are available in adults, they remain to be investigated in forthcoming studies. In view of the fact that ADMA values tend to be higher in neonates and young children [44,45], we hypothesize that the lack of association between CKD staging and ADMA concentrations is because of the predominance of younger children in early stages of CKD and small sample size without ability to perform age subgroup analysis. Additionally, simultaneous ADMA measurements in blood and urine are incomplete in most studies, which might help identify their importance in pathophysiology. Taking all these together, ADMA is an emerging as a diagnostic and prognostic biomarker in certain pediatric kidney diseases. Despite plasma and urinary ADMA are considered cardiovascular risk factors in adults [5,50], such evidence is still slim in children and adolescents. Additionally, its role as a predictive biomarker in pediatric kidney disease has not been examined yet.

## 4. ADMA as a Therapeutic Target

However, is ADMA just a risk biomarker, or does it play a crucial role in the pathogenesis of pediatric kidney disease? Emerging evidence supports the view that countering ADMA may be a relevant, worthy area of intervention to prevent CVD and CKD progression [6,21,24]. As reviewed elsewhere [1,16,19,21], several drugs have shown ADMA-lowering effects in clinical studies. These include fenofibrate, angiotensin-converting enzyme inhibitors, angiotensin receptor blockers, metformin, folic acid, α-lipoic acid, and oral contraceptives. However, the mechanism behind these drugs in reducing ADMA may still not be clear.

Considering almost 80% of ADMA is degraded in the body, therapeutic approaches have been assessed to enhance its degradation via enhancing DDAH enzymes and/or activity. Today, several therapies have been shown to increase DDAH enzymes and/or activity and thereby diminish ADMA concentrations in a broad range of animal models. The list of reported medication consisted of melatonin [57], farnesoid X receptor agonist [76], pioglitazone [77], telmisartan [78], aliskiren [79], N-acetylcysteine [80], metformin [81], vitamin E [82], and shichimotsukokato [83].

On the other hand, epigallocatechin-3-gallate [84], glucagon-like peptide-1 receptor agonist [85], and telmisartan [78] were shown to lower ADMA levels coinciding with downregulation of PRMT-1 expression. The recent discovery of high-resolution crystal structures of DDAH isoforms provides an insight into the molecular mechanisms that regulate their activities [86]. There is a clear need to move beyond these studies to develop pharmacological and biological agents modulating DDAHs and/or PRMTs in the near future.

## 5. Conclusions and Future Perspectives

Today, the measurement of ADMA in blood or urine is a useful measure of L-arginine/ADMA/NO pathway in kidney disease. Considering that ADMA levels are much greater in neonates and children than in adults, there may be additional NO-independent effects of ADMA in the pediatric population. ADMA has been used as a biomarker for diagnosis and prognosis in pediatric renal diseases and has helped identify its importance in pathophysiology, while numerous challenges remain to be overcome in translating biomarker research into the clinical space.

Discrepancies observed among various studies for reported ADMA values could be related to age [43,44,45], body mass [40], race [43], or methodology [40]. Heterogeneity is an unavoidable feature in pediatric research, especially in biomarker interpretation. From birth to adolescents, many physiological alterations occur in the body. ADMA concentration is greatly variable in the pediatric population due to the broad age range. Additionally, standardization of methodological heterogeneity may help to gain insight into comparisons between different studies and yield definite conclusions. Currently, various methods have been established for the measurement of ADMA. Nevertheless, most methods have obvious limitations, especially performed on a routine basis in the clinical setting. Therefore, future work in developing a simple high-precision method for measuring ADMA in clinical practice is a necessity to advance our knowledge of the role of ADMA as a biomarker in many pediatric disorders.

Another important aspect is that most pediatric studies have been limited by a small sample size and inadequate power. Although substantial evidence indicates a direct association between ADMA and CV risk in adult patients with CKD, such evidence is still lacking in the pediatric population. Thus, large multicenter studies regarding kidney diseases are needed to be able to establish more robust true relationships in children and adolescents.

Pharmacological studies aiming to modulate the activity of DDAH/PRMT are also relatively rare, especially in the pediatric population. It is imperative that specific ADMA-lowering agents still require investigation. It is expected that the measurement of not just ADMA but even ADMA-related indices that represent whole-body ADMA synthesis or DDAH activity will give rise to valuable information, resulting in a more complete picture and understanding of the involved pathways in various pediatric renal diseases.

In summary, the aim of this review has been to point to some of the steps in these processes that would benefit from further work to illuminate the role of ADMA as a biomarker and to perhaps explore its significance in pathophysiology in pediatric renal diseases.

## Figures and Tables

**Table 1:** Concentrations of ADMA in children and adolescents with kidney disease.

Type of Kidney Disease	Study	Group of Patients	Age, Years	ADMA	Analytic Method	Ref.
	Plasma level (μM/L)					
CKD	Brook et al., 2009	28 CKD stage 2–3 cases 10 controls	12.6 ± 1 11.3 ± 4.7	1.1 ± 0.3 * 0.8 ± 0.2	HPLC–MS	[61,62]
	Chien et al., 2015	34 CKD stage 1 cases 23 CKD stage 2–3 cases	8.5 (6.1–13.9) 14.7 (9.6–16.8)	0.8 (0.5–1.6) 0.6 (0.4–1.2)	HPLC	[63]
	Hsu et al., 2019	74 CKD stage 1–4 with normal BP cases 47 CKD stage 1–4 with abnormal BP cases	10 (6.4–14.2) 8.7 (4.8–15.7)	1.05 (0.7–1.33) 1.1 (0.8–1.3)	HPLC	[64]
	Benito et al., 2018	24 controls 32 CKD stage 2–5 cases	6–18 3–17	0.7 (0.2–1.1) 0.9 (0.6–1.4)	LC–MS	[65]
	Makulska et al., 2015	26 controls 36 CKD cases 20 PD cases 20 HD cases	14.5 ± 3.3 14.9 ± 3.5 14.3 ± 2.3 15 ± 3.3	0.39 ± 0.01 0.65 ± 0.03 * 0.78 ± 0.01 * 0.85 ± 0.01 *	ELISA	[66]
	Snauwaert et al., 2018	50 controls 57 CKD stage 1–5 cases	6.7 (4.2–9.8) 8.8 (5.1–14.7)	0.67 ± 0.11 NS	ELISA	[67]
FSGS	Lücke et al., 2008	9 FSGS cases 11 non-FSGS cases 9 controls	5–18	0.85 ± 0.11 * 0.79 ± 0.13 0.68 ± 0.11	GC–MS/MS	[68]
INS	Hyla-Klekot et al., 2015	32 INS cases at relapse 32 INS cases at remission	2–17	0.53 ± 0.11 0.54 ± 0.11	HPLC	[69]
Glomerular kidney disease	Skrzypczyk et al., 2019	42 INS cases 38 IgAN/HSN cases	10.8 ±4.4 11.9 ±4.1	1.72 ± 1.24 1.6 ± 1.19	ELISA	[70]
HUS		12 HUS received PD cases 12 controls	3.6 ± 3.5	0.67 ± 0.16 0.75 ±0.21	GC–MS/MS	[71]
	Urine (μM/mM creatinine)					
CKD	Kuo et al., 2012	20 CKD stage 1 cases 25 CKD stage 2–4 cases	13 (5–18) 15 (5–18)	3.1 (0.4–20.8) 1.9 (0.1–9.4)	HPLC	[72]
	Lin et al., 2016	33 CKD stage 1 cases 22 CKD stage 2–3 cases	8.6 (6.6–14) 14.9 (11.4–16.8)	16.9 (11.1–32.1) 16.5 (11.1–26.1)	HPLC	[73]
FSGS	Lücke et al., 2008	9 FSGS cases 11 non-FSGS cases 9 controls	5–18	41.4 ± 5.5 NS 15.7 ± 2.6	GC–MS/MS	[68]
HUS		5 HUS received PD cases 9 controls	3.6 ± 3.5	3.3 ± 2.5 * 10.1 ± 6.5	GC–MS/MS	[71]

Data on age and ADMA levels are presented as mean ± standard deviation or median (interquartile range); ADMA = Asymmetric dimethylarginine; CKD = Chronic kidney disease; PD = Peritoneal dialysis; HD = Hemodialysis; FSGS = Focal segmental glomerulosclerosis; INS = Idiopathic nephrotic syndrome, IgAN = IgA nephropathy; HSN = Henoch-Schoenlein nephropathy; HUS = hemolytic uremic syndrome; NS = Not shown; * *p* < 0.05 versus controls.

## Data Availability

Data is contained within the article.

## References

[1] Tain Y.L., Hsu C.N. (2017). Toxic Dimethylarginines: Asymmetric Dimethylarginine (ADMA) and Symmetric Dimethylarginine (SDMA). Toxins.

[2] Vallance P., Leone A., Calver A., Collier J., Moncada S. (1992). Accumulation of an endogenous inhibitor of nitric oxide synthesis in chronic renal failure. Lancet.

[3] Cooke J.P. (2000). Does ADMA cause endothelial dysfunction?. Arterioscler. Thromb. Vasc. Biol..

[4] Leiper J., Nandi M. (2011). The therapeutic potential of targeting endogenous inhibitors of nitric oxide synthesis. Nat. Rev. Drug Discov..

[5] Tripepi G., Mattace Raso F., Sijbrands E., Seck M.S., Maas R., Boger R., Witteman J., Rapisarda F., Malatino L., Mallamaci F. (2011). Inflammation and asymmetric dimethylarginine for predicting death and cardiovascular events in ESRD patients. Clin. J. Am. Soc. Nephrol..

[6] Ueda S., Yamagishi S., Okuda S. (2010). New pathways to renal damage: Role of ADMA in retarding renal disease progression. J. Nephrol..

[7] Kakimoto Y., Akazawa S. (1970). Isolation and identification of N-^G^,N-^G^- and N-^G^,N’-^G^-dimethyl-arginine, N-epsilon-mono-, di-, and trimethyllysine, and glucosylgalactosyl- and galactosyl-delta-hydroxylysine from human urine. J. Biol. Chem..

[8] McDermott J.R. (1976). Studies on the catabolism of Ng-methylarginine, Ng, Ng-dimethylarginine and Ng, Ng-dimethylarginine in the rabbit. Biochem. J..

[9] Ogawa T., Kimoto M., Sasaoka K. (1987). Occurrence of a new enzyme catalyzing the direct conversion of N^G^,N^G^-dimethyl-L-arginine to L-citrulline in rats. Biochem. Biophys. Res. Commun..

[10] Leiper J.M., Santa Maria J., Chubb A., MacAllister R.J., Charles I.G., Whitley G.S., Vallance P. (1999). Identification of two human dimethylarginine dimethylaminohydrolases with distinct tissue distributions and homology with microbial arginine deiminases. Biochem. J..

[11] Rodionov R.N., Martens-Lobenhoffer J., Brilloff S., Hohenstein B., Jarzebska N., Jabs N., Kittel A., Maas R., Weiss N., Bode-Böger S.M. (2014). Role of alanine:glyoxylate aminotransferase 2 in metabolism of asymmetric dimethylarginine in the settings of asymmetric dimethylarginine overload and bilateral nephrectomy. Nephrol. Dial. Transplant..

[12] Park M.J., Oh K.S., Nho J.H., Kim G.Y., Kim D.I. (2016). Asymmetric dimethylarginine (ADMA) treatment induces apoptosis in cultured rat mesangial cells via endoplasmic reticulum stress activation. Cell Biol. Int..

[13] Sydow K., Münzel T. (2003). ADMA and oxidative stress. Atheroscler. Suppl..

[14] Shirakawa T., Kako K., Shimada T., Nagashima Y., Nakamura A., Ishida J., Fukamizu A. (2011). Production of free methylarginines via the proteasome and autophagy pathways in cultured cells. Mol. Med. Rep..

[15] Zheng N., Wang K., He J., Qiu Y., Xie G., Su M., Jia W., Li H. (2016). Effects of ADMA on gene expression and metabolism in serum-starved LoVo cells. Sci. Rep..

[16] Tain Y.L., Hsu C.N. (2016). Targeting on Asymmetric dimethylarginine-related nitric oxide-reactive oxygen species imbalance to reprogram the development of hypertension. Int. J. Mol. Sci..

[17] Schepers E., Barreto D.V., Liabeuf S., Glorieux G., Eloot S., Barreto F.C., Massy Z., Vanholder R., European Uremic Toxin Work Group (EUTox) (2011). Symmetric dimethylarginine as a proinflammatory agent in chronic kidney disease. Clin. J. Am. Soc. Nephrol..

[18] Pekarova M., Kubala L., Martiskova H., Bino L., Twarogova M., Klinke A., Rudolph T.K., Kuchtova Z., Kolarova H., Ambrozova G. (2013). Asymmetric dimethylarginine regulates the lipopolysaccharide- induced nitric oxide production in macrophages by suppressing the activation of NF-kappaB and iNOS expression. Eur. J. Pharmacol..

[19] Tain Y.L., Huang L.T. (2011). Asymmetric dimethylarginine: Clinical applications in pediatric medicine. J. Formos. Med. Assoc..

[20] Kielstein J.T., Fliser D. (2007). The past, presence and future of ADMA in nephrology. Nephrol. Ther..

[21] Bełtowski J., Kedra A. (2006). Asymmetric dimethylarginine (ADMA) as a target for pharmacotherapy. Pharmacol. Rep..

[22] Tain Y.L., Huang L.T. (2014). Restoration of asymmetric dimethylarginine-nitric oxide balance to prevent the development of hypertension. Int. J. Mol. Sci..

[23] Morales Y., Cáceres T., May K., Hevel J.M. (2016). Biochemistry and regulation of the protein arginine methyltransferases (PRMTs). Arch. Biochem. Biophys..

[24] Teerlink T., Luo Z., Palm F., Wilcox C.S. (2009). Cellular ADMA: Regulation and action. Pharmacol. Res..

[25] Bode-Böger S.M., Scalera F., Ignarro L.J. (2007). The L-arginine paradox: Importance of the L-arginine/asymmetrical dimethylarginine ratio. Pharmacol. Ther..

[26] Palm F., Onozato M.L., Luo Z., Wilcox C.S. (2007). Dimethylarginine dimethylaminohydrolase (DDAH): Expression, regulation, and function in the cardiovascular and renal systems. Am. J. Physiol. Heart Circ. Physiol..

[27] Sorrenti V., Mazza F., Campisi A., Vanella L., Li V.G., Di G.C. (2006). High glucose-mediated imbalance of nitric oxide synthase and dimethylarginine dimethylaminohydrolase expression in endothelial cells. Curr. Neurovasc. Res..

[28] Brands M.W., Bell T.D., Gibson B. (2004). Nitric oxide may prevent hypertension early in diabetes by counteracting renal actions of superoxide. Hypertension.

[29] Tain Y.L., Kao Y.H., Hsieh C.S., Chen C.C., Sheen J.M., Lin I.C., Huang L.T. (2010). Melatonin blocks oxidative stress-induced increased asymmetric dimethylarginine. Free Radic. Biol. Med..

[30] Saigusa D., Takahashi M., Kanemitsu Y., Ishida A., Abe T., Yamakuni T., Suzuki N., Tomioka Y. (2011). Determination of Asymmetric Dimethylarginine and Symmetric Dimethylarginine in Biological Samples of Mice Using LC/MS/MS. Am. J. Anal. Chem..

[31] Wang D., Gill P.S., Chabrashvili T., Onozato M.L., Raggio J., Mendonca M., Dennehy K., Li M., Modlinger P., Leiper J. (2007). Isoform-specific regulation by N^G^,N^G^-dimethylarginine dimethylaminohydrolase of rat serum asymmetric dimethylarginine and vascular endothelium-derived relaxing factor/NO. Circ. Res..

[32] Wilcken D.E., Sim A.S., Wang J., Wang X.L. (2007). Asymmetric dimethylarginine (ADMA) in vascular, renal and hepatic disease and the regulatory role of L-arginine on its metabolism. Mol. Genet. Metab..

[33] Tsikas D. (2008). A critical review and discussion of analytical methods in the L-arginine/nitric oxide area of basic and clinical research. Anal. Biochem..

[34] Teerlink T., Nijveldt R.J., de Jong S., van Leeuwen P.A.M. (2002). Determination of arginine, asymmetric dimethylarginine, and symmetric dimethylarginine in human plasma and other biological samples by high-performance liquid chromatography. Anal. Biochem..

[35] Boelaert J., Schepers E., Glorieux G., Eloot S., Vanholder R., Lynen F. (2016). Determination of Asymmetric and Symmetric Dimethylarginine in Serum from Patients with Chronic Kidney Disease: UPLC-MS/MS versus ELISA. Toxins.

[36] Martens-Lobenhoffer J., Krug O., Bode-Boger S.M. (2004). Determination of arginine and asymmetric dimethylarginine (ADMA) in human plasma by liquid chromatography/mass spectrometry with the isotope dilution technique. J. Mass Spectrom..

[37] Hui Y., Wong M., Kim J.-O., Love J., Ansley D.M., Chen D.D.Y. (2012). A new derivatization method coupled with LC-MS/MS to enable baseline separation and quantification of dimethylarginines in human plasma from patients to receive on-pump CABG surgery. Electrophoresis.

[38] Tsikas D., Beckmann B., Gutzki F.M., Jordan J. (2011). Simultaneous gas chromatography-tandem mass spectrometry quantification of symmetric and asymmetric dimethylarginine in human urine. Anal. Biochem..

[39] Schulze F., Wesemann R., Schwedhelm E., Sydow K., Albsmeier J., Cooke J.P., Böger R.H. (2004). Determination of asymmetric dimethylarginine (ADMA) using a novel ELISA assay. Clin. Chem. Lab. Med..

[40] Horowitz J.D., Heresztyn T. (2007). An overview of plasma concentrations of asymmetric dimethylarginine (ADMA) in health and disease and in clinical studies: Methodological considerations. J. Chromatogr. B Anal. Technol. Biomed. Life Sci..

[41] Martens-Lobenhoffer J., Westphal S., Awiszus F., Bode-Boger S.M., Luley C. (2005). Determination of asymmetric dimethylarginine: Liquid chromatography-mass spectrometry or ELISA?. Clin. Chem..

[42] Németh B., Ajtay Z., Hejjel L., Ferenci T., Ábrám Z., Murányi E., Kiss I. (2017). The issue of plasma asymmetric dimethylarginine reference range—A systematic review and meta-analysis. PLoS ONE.

[43] Sydow K., Fortmann S.P., Fair J.M., Varady A., Hlatky M.A., Go A.S., Iribarren C., Tsao P.S., ADVANCE Investigators (2010). Distribution of asymmetric dimethylarginine among 980 healthy, older adults of different ethnicities. Clin. Chem..

[44] Lücke T., Kanzelmeyer N., Kemper M.J., Tsikas D., Das A.M. (2007). Developmental changes in the Larginine/nitric oxide pathway from infancy to adulthood: Plasma asymmetric dimethylarginine levels decrease with age. Clin. Chem. Lab. Med..

[45] Vida G., Sulyok E., Ertl T., Martens-Lobenhoffer J., Bode-Boger S.M. (2007). Plasma asymmetric dimethylarginine concentration during the perinatal period. Neonatology.

[46] Vanholder R., De Smet R., Glorieux G., Argilés A., Baurmeister U., Brunet P., Clark W., Cohen G., De Deyn P.P., Deppisch R. (2003). Review on uremic toxins: Classification, concentration, and interindividual variability. Kidney Int..

[47] Schepers E., Speer T., Bode-Böger S.M., Fliser D., Kielstein J.T. (2014). Dimethylarginines ADMA and SDMA: The Real Water-Soluble Small Toxins?. Semin. Nephrol..

[48] Anderstam B., Katzarski K., Bergstrom J. (1997). Serum levels of NG, NG-dimethyl-L-arginine, a potential endogenous nitric oxide inhibitor in dialysis patients. J. Am. Soc. Nephrol..

[49] Jacobi J., Tsao P.S. (2008). Asymmetrical dimethylarginine in renal disease: Limits of variation or variation limits? A systematic review. Am. J. Nephrol..

[50] Schlesinger S., Sonntag S.R., Lieb W., Maas R. (2016). Asymmetric and symmetric dimethylarginine as risk markers for total mortality and cardiovascular outcomes: A systematic review and meta-analysis of prospective studies. PLoS ONE.

[51] Kone B.C. (2004). Nitric oxide synthesis in the kidney: Isoforms, biosynthesis, and functions in health. Semin. Nephrol..

[52] Hsu C.N., Tain Y.L. (2019). Regulation of nitric oxide production in the developmental programming of hypertension and kidney disease. Int. J. Mol. Sci..

[53] Hsu C.N., Huang L.T., Lau Y.T., Lin C.Y., Tain Y.L. (2012). The combined ratios of L-arginine and asymmetric and symmetric dimethylarginine as biomarkers in spontaneously hypertensive rats. Transl. Res..

[54] Tsai C.M., Kuo H.C., Hsu C.N., Huang L.T., Tain Y.L. (2014). Metformin reduces asymmetric dimethylarginine and prevents hypertension in spontaneously hypertensive rats. Transl. Res..

[55] Li Volti G., Salomone S., Sorrenti V., Mangiameli A., Urso V., Siarkos I., Galvano F., Salamone F. (2011). Effect of silibinin on endothelial dysfunction and ADMA levels in obese diabetic mice. Cardiovasc. Diabetol..

[56] Tain Y.L., Lee W.C., Hsu C.N., Lee W.C., Huang L.T., Lee C.T., Lin C.Y. (2013). Asymmetric dimethylarginine is associated with developmental programming of adult kidney disease and hypertension in offspring of streptozotocin-treated mothers. PLoS ONE.

[57] Tain Y.L., Hsieh C.S., Chen C.C., Sheen J.M., Lee C.T., Huang L.T. (2010). Melatonin prevents increased asymmetric dimethylarginine in young rats with bile duct ligation. J. Pineal Res..

[58] Sheen J.M., Huang L.T., Hsieh C.S., Chen C.C., Wang J.Y., Tain Y.L. (2010). Bile duct ligation in developing rats: Temporal progression of liver, kidney, and brain damage. J. Pediatr. Surg..

[59] Tain Y.L., Hsu C.N., Chan J.Y., Huang L.T. (2015). Renal transcriptome analysis of programmed hypertension induced by maternal nutritional insults. Int. J. Mol. Sci..

[60] Tain Y.L., Huang L.T., Chan J.Y., Lee C.T. (2015). Transcriptome analysis in rat kidneys: Importance of genes involved in programmed hypertension. Int. J. Mol. Sci..

[61] Wang S., Vicente F.B., Miller A., Brooks E.R., Price H.E., Smith F.A. (2007). Measurement of arginine derivatives in pediatric patients with chronic kidney disease using high-performance liquid chromatography-tandem mass spectrometry. Clin. Chem. Lab. Med..

[62] Brooks E.R., Langman C.B., Wang S., Price H.E., Hodges A.L., Darling L., Yang A.Z., Smith F.A. (2009). Methylated arginine derivatives in children and adolescents with chronic kidney disease. Pediatr. Nephrol..

[63] Chien S.J., Lin I.C., Hsu C.N., Lo M.H., Tain Y.L. (2015). Homocysteine and arginine-to-asymmetric dimethylarginine ratio associated with blood pressure abnormalities in children with early chronic kidney disease. Circ. J..

[64] Hsu C.N., Lu P.C., Lo M.H., Lin I.C., Tain Y.L. (2019). The association between nitric oxide pathway, blood pressure abnormalities, and cardiovascular risk profile in pediatric chronic kidney disease. Int. J. Mol. Sci..

[65] Benito S., Sánchez-Ortega A., Unceta N., Jansen J.J., Postma G., Andrade F., Aldámiz-Echevarria L., Buydens L.M.C., Goicolea M.A., Barrio R.J. (2018). Plasma biomarker discovery for early chronic kidney disease diagnosis based on chemometric approaches using LC-QTOF targeted metabolomics data. J. Pharm. Biomed. Anal..

[66] Makulska I., Szczepańska M., Drożdż D., Polak-Jonkisz D., Zwolińska D. (2015). Skin autofluorescence as a novel marker of vascular damage in children and adolescents with chronic kidney disease. Pediatr. Nephrol..

[67] Snauwaert E., Van Biesen W., Raes A., Glorieux G., Van Bogaert V., Van Hoeck K., Coppens M., Roels S., Vande Walle J., Eloot S. (2018). Concentrations of representative uraemic toxins in a healthy versus non-dialysis chronic kidney disease paediatric population. Nephrol. Dial. Transplant..

[68] Lücke T., Kanzelmeyer N., Chobanyan K., Tsikas D., Franke D., Kemper M.J., Ehrich J.H., Das A.M. (2008). Elevated asymmetric dimethylarginine (ADMA) and inverse correlation between circulating ADMA and glomerular filtration rate in children with sporadic focal segmental glomerulosclerosis (FSGS). Nephrol. Dial. Transplant..

[69] Hyla-Klekot L., Bryniarski P., Pulcer B., Ziora K., Paradysz A. (2015). Dimethylarginines as risk markers of atherosclerosis and chronic kidney disease in children with nephrotic syndrome. Adv. Clin. Exp. Med..

[70] Skrzypczyk P., Przychodzień J., Mizerska-Wasiak M., Kuźma-Mroczkowska E., Stelmaszczyk-Emmel A., GóRSKA E., Pańczyk-Tomaszewska M. (2019). Asymmetric dimethylarginine is not a marker of arterial damage in children with glomerular kidney diseases. Cent. Eur. J. Immunol..

[71] Kanzelmeyer N.K., Pape L., Chobanyan-Jürgens K., Tsikas D., Hartmann H., Fuchs A.J., Vaske B., Das A.M., Haubitz M., Jordan J. (2014). L-arginine/NO pathway is altered in children with haemolytic-uraemic syndrome (HUS). Oxid. Med. Cell Longev..

[72] Kuo H.C., Hsu C.N., Huang C.F., Lo M.H., Chien S.J., Tain Y.L. (2012). Urinary arginine methylation index associated with ambulatory blood pressure abnormalities in children with chronic kidney disease. J. Am. Soc. Hypertens..

[73] Lin I.C., Hsu C.N., Lo M.H., Chien S.J., Tain Y.L. (2016). Low urinary citrulline/arginine ratio associated with blood pressure abnormalities and arterial stiffness in childhood chronic kidney disease. J. Am. Soc. Hypertens..

[74] Tsikas D. (2020). Urinary dimethylamine (DMA) and its precursor asymmetric dimethylarginine (ADMA) in clinical medicine, in the context of nitric oxide (NO) and beyond. J. Clin. Med..

[75] Hsu C.N., Chang-Chien G.P., Lin S., Hou C.Y., Ku P.C., Tain Y.L. (2020). Association of trimethylamine, trimethylamine N-oxide, and dimethylamine with cardiovascular risk in children with chronic kidney disease. J. Clin. Med..

[76] Hu T., Chouinard M., Cox A.L., Sipes P., Marcelo M., Ficorilli J., Li S., Gao H., Ryan T.P., Michael M.D. (2006). Farnesoid X receptor agonist reduces serum asymmetric dimethylarginine levels through hepatic dimethylarginine dimethylaminohydrolase-1 gene regulation. J. Biol. Chem..

[77] Wakino S., Hayashi K., Tatematsu S., Hasegawa K., Takamatsu I., Kanda T., Homma K., Yoshioka K., Sugano N., Saruta T. (2005). Pioglitazone lowers systemic asymmetric dimethylarginine by inducing dimethylarginine dimethylaminohydrolase in rats. Hypertens. Res..

[78] Onozato M.L., Tojo A., Leiper J., Fujita T., Palm F., Wilcox C.S. (2008). Expression of NG,NG-dimethylarginine dimethylaminohydrolase and protein arginine N-methyltransferase isoforms in diabetic rat kidney: Effects of angiotensin II receptor blockers. Diabetes.

[79] Tain Y.L., Hsu C.N., Lin C.Y., Huang L.T., Lau Y.T. (2011). Aliskiren prevents hypertension and reduces asymmetric dimethylarginine in young spontaneously hypertensive rats. Eur. J. Pharmacol..

[80] Fan N.C., Tsai C.M., Hsu C.N., Huang L.T., Tain Y.L. (2013). N-acetylcysteine prevents hypertension via regulation of the ADMA-DDAH pathway in young spontaneously hypertensive rats. Biomed. Res. Int..

[81] Tain Y.L., Huang L.T., Hsu C.N., Lee C.T. (2014). Melatonin therapy prevents programmed hypertension and nitric oxide deficiency in offspring exposed to maternal caloric restriction. Oxid. Med. Cell Longev..

[82] Yang Y.Y., Lee T.Y., Huang Y.T., Chan C.C., Yeh Y.C., Lee F.Y., Lee S.D., Lin H.C. (2012). Asymmetric dimethylarginine (ADMA) determines the improvement of hepatic endothelial dysfunction by vitamin E in cirrhotic rats. Liver Int..

[83] Bai F., Makino T., Ono T., Mizukami H. (2012). Anti-hypertensive effects of shichimotsukokato in 5/6 nephrectomized Wistar rats mediated by the DDAH-ADMA-NO pathway. J. Nat. Med..

[84] Chen D., Zhang K.Q., Li B., Sun D.Q., Zhang H., Fu Q. (2016). Epigallocatechin-3-gallate ameliorates erectile function in aged rats via regulation of PRMT1/DDAH/ADMA/NOS metabolism pathway. Asian J. Androl..

[85] Ojima A., Ishibashi Y., Matsui T., Maeda S., Nishino Y., Takeuchi M., Fukami K., Yamagishi S. (2013). Glucagon-like peptide-1 receptor agonist inhibits asymmetric dimethylarginine generation in the kidney of streptozotocin-induced diabetic rats by blocking advanced glycation end product-induced protein arginine methyltranferase-1 expression. Am. J. Pathol..

[86] Wadham C., Mangoni A.A. (2009). Dimethylarginine dimethylaminohydrolase regulation: A novel therapeutic target in cardiovascular disease. Expert Opin. Drug Metab. Toxicol..

